# The Effects of Live Transport on Metabolism and Stress Responses of Abalone (*Haliotis iris*)

**DOI:** 10.3390/metabo11110748

**Published:** 2021-10-29

**Authors:** Andrea C. Alfaro, Thao V. Nguyen, Leonie Venter, Jessica A. Ericson, Shaneel Sharma, Norman L. C. Ragg, Craig Mundy

**Affiliations:** 1 Aquaculture Biotechnology Research Group, School of Science, Auckland University of Technology, Private Bag 92006, Auckland 1142, New Zealand; thao.vanguyen@gmail.com (T.V.N.); leonie.venter@aut.ac.nz (L.V.); shaneel.sharma@aut.ac.nz (S.S.); 2 NTT Hi-Tech Institute, Nguyen Tat Thanh University, Ho Chi Minh City 755414, Vietnam; 3 Cawthron Institute, Private Bag 2, Nelson 7042, New Zealand; jess.ericson@cawthron.org.nz (J.A.E.); norman.ragg@cawthron.org.nz (N.L.C.R.); 4 IMAS Fisheries and Aquaculture Centre, College of Science and Engineering, University of Tasmania, Taroona, Hobart 7001, Australia; craig.mundy@utas.edu.au

**Keywords:** metabolomics, abalone, stress responses, live transportation, resuscitation, GC–MS

## Abstract

The New Zealand abalone industry relies mostly on the export of processed products to distant Asian markets, notably China. Over the past five years, live export of high quality abalone from New Zealand has proven successful. However, transport of live animals is associated with multiple stressors that affect survival and meat quality at the end of the transport phase. Better understanding of transport-derived stress is needed to improve transport conditions and recovery at destination to ensure high product quality and safety throughout the supply chain. To this end, we applied an untargeted GC–MS-based metabolomics approach to examine the changes in metabolite profiles of abalone after a 2-day transport event and subsequent water re-immersion for 2 days. The results revealed alterations of many metabolites in the haemolymph and muscle of post-transported abalone. Decreased concentrations of many amino acids suggest high energy demands for metabolism and stress responses of transported abalone, while increases of other amino acids may indicate active osmoregulation and/or protein degradation due to oxidative stress and apoptosis. The accumulation of citric acid cycle intermediates and anaerobic end-products are suggestive of hypoxia stress and a shift from aerobic to anaerobic metabolism (resulting from aerial exposure). Interestingly, some features in the metabolite profile of reimmersed abalone resembled those of pre-transported individuals, suggesting progressive recovery after reimmersion in water. Evidence of recovery was observed in the reduction of some stress biomarkers (e.g., lactic acid, succinic acid) following reimmersion. This study revealed insights into the metabolic responses to transport stress in abalone and highlights the importance of reimmersion practices in the supply chain of live animal exports.

## 1. Introduction

There are approximately 14 abalone species currently harvested from the wild or produced through aquaculture for human consumption [1,2,3,4]. The total abalone production in 2019 was 210,926 metric tonnes (mt), of which 96.4% was from aquaculture [3,4]. Australia is currently the leading producer of wild-caught abalone in the world, while China represents the world’s largest supplier of farmed abalone [3,4]. New Zealand is also an important abalone producer with 730 mt in 2018, mostly from one species (*Haliotis Iris* Gmelin, 1791), of which 87% comes from fisheries and the remaining quantity comes from a growing aquaculture industry (MPI, 2019). New Zealand exported 145 mt of abalone products valued at 7.7 million NZD in 2020 [5]. New Zealand abalone are sold as live, fresh or chilled, frozen, processed and preserved products [5], with live exports (packed in cooled polystyrene boxes without water) accounting for 43% of total exports [5]. Transport by air freight can take up to 36 h depending on the destination. For live export, animals are often held in seawater post-harvest for up to three weeks before transport and then re-immersed in seawater upon arrival at the destination and before distribution to local markets.

Live transport is known to result in animal stress, compromised meat quality, morbidity and mortality that may lead to significant economic loses [6,7]. Stress conditions for transported shellfish may include suboptimal transport environments (e.g., temperature shifts, low humidity, aerial exposure, low oxygen and overcrowding) and physical manipulation (e.g., capture, cleaning and handling) [6]. Attempts to reduce these adverse effects have resulted in various transport modified environments, such as oxygen-enriched dry containers maintained at low temperatures or cooled polystyrene boxes for short-term transport. While transport of shellfish in water can be achieved in simple water containers with oxygen supply and water treatment systems [8], the cost of freighting water is prohibitively high, and mechanisms for the return of containers are expensive and pose biosecurity risks. Thus, a better understanding of the mechanisms involved in transport stress responses, especially at the molecular level, would provide useful information for improving transport methods, animal health and meat quality. A number of studies have investigated the physiological and immunological responses of shellfish associated with live transport (reviewed by Fotedar and Evans [6]). Many of these investigated the responses of abalone to handling [9], air exposure [10,11], oxygen and temperature stress [12] and contribution of anaerobic metabolism to the energetic cost of transport [13].

Metabolomics has been increasingly applied in aquatic research to elucidate the effects of exogenous and endogenous perturbations on marine organisms under various exposure conditions [14,15,16,17,18,19]. Through this approach, metabolites (low weight molecules found in cells, tissues and organisms) can be profiled to identify changes in physiological, metabolic and developmental state under specific conditions to reveal mechanistic information as well as biomarker identification. A number of recent studies have specifically demonstrated the successful application of metabolomics to understand the effects of different external stressors and conditions on shellfish, such as pathogen infections and diseases [20,21,22,23,24], functional feeds (e.g., probiotics, prebiotics) [25,26,27], water contaminants [28,29], and harvesting, transporting and storing effects [7,30]. In addition, many candidate biomarkers have been identified for these conditions (such as itaconic acid, lactic acid and succinic acid). Recently, metabolomics approaches have also been applied to understand the molecular mechanisms underlying the response of shellfish to harvesting, storage and transport [7,30]. However, such studies are limited for abalone [19]. In this paper, we employed a GC–MS-based metabolomics approach to identify the effects of simulated transport stress on New Zealand black-footed abalone (*H. iris*; locally known as “pāua”) at three different sampling points (pre-transport, 2 days post-transport and 2 days after re-immersion in water following transport). It is envisaged that this information will be useful to improve live abalone transport and recovery methods leading to higher survival rates and improved product quality.

## 2. Results

### 2.1. Metabolic Responses in Haemolymph

**Figure 1:**
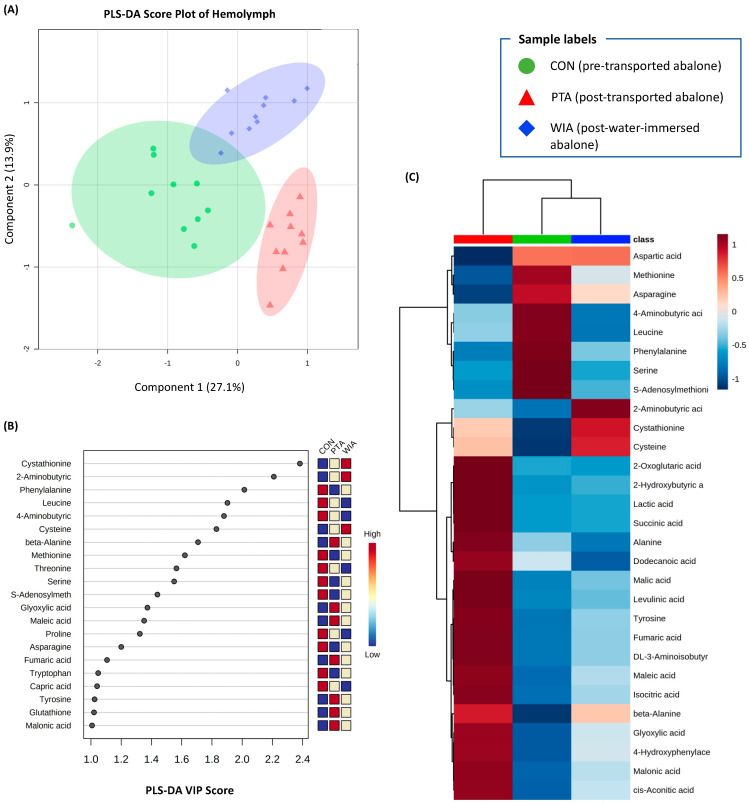
Effects of transport and re-immersion on normalised metabolite profiles of abalone haemolymph. (**A**) PLS-DA score plots. (**B**) Relative abundance of metabolites with VIP scores greater than 1.0. (**C**) Heatmap of 29 metabolites that differed among treatments. The colour bars in the PLS-DA VIP score plot and heatmap show the relative abundance of metabolites (stronger blue: lower abundance; stronger red: higher abundance).

We identified 68 annotated metabolites in spectra of abalone haemolymph. The majority of these metabolites were amino acids, fatty acids and organic compounds. The multivariate data analyses with partial least squares-discriminant analysis (PLS-DA) revealed good separation among the three treatment groups (control/pre-transport [CON], post-transported abalone (PTA) and post-water-immersed abalone (WIA)) (Figure 1A). The first two components made up of 35.9% of the total variation. The cross validation of PLS-DA model via leave-one-out cross-validation (LOOCV) of the first 5 components showed very high scores of accuracy (1.0), multiple correlation coefficients (R^2^, 0.94) and cross-validated R^2^ (Q^2^, 0.67), indicating good fitness and prediction performance of the PLS-DA model. PLS-DA variable importance in projection (VIP) scores identified 21 metabolites with VIP scores greater than 1, which were important classifiers (Figure 1B).

A one-way ANOVA identified 29 metabolites that were significantly different among the treatments. A heatmap showed a relative abundance of these altered metabolites in each treatment (Figure 1C). Compared to control abalone, post-transported animals showed decreases in many amino acids (e.g., aspartic acid, methionine, asparagine), and increases in other compounds, such as amino acids (e.g., cystathionine, cysteine), citric acid (TCA) cycle intermediates (e.g., succinic acid, isocitric acid, malic acid) and other organic compounds (e.g., lactic acid, maleic acid). The metabolite profile of post-water-immersed abalone resembled the control group, but, notably, had different levels of many metabolites (Figure 1C).

Pathway analysis of haemolymph metabolite profiles of post-transported abalone compared to control (pre-transport) abalone identified 16 pathways of interest with pathway impact scores (PIs) greater than 0.01 (Table 1). Among these, only six pathways were significantly affected by transport (*p* < 0.05) (cysteine and methionine metabolism; glycine, serine and threonine metabolism; phenylalanine metabolism; phenylalanine, tyrosine and tryptophan biosynthesis; tyrosine metabolism; glyoxylate and dicarboxylate metabolism). The remaining 12 pathways (*p* > 0.05) were considered to be only slightly affected by the transport event (Table 1).

### 2.2. Metabolic Responses in Muscle

**Figure 2:**
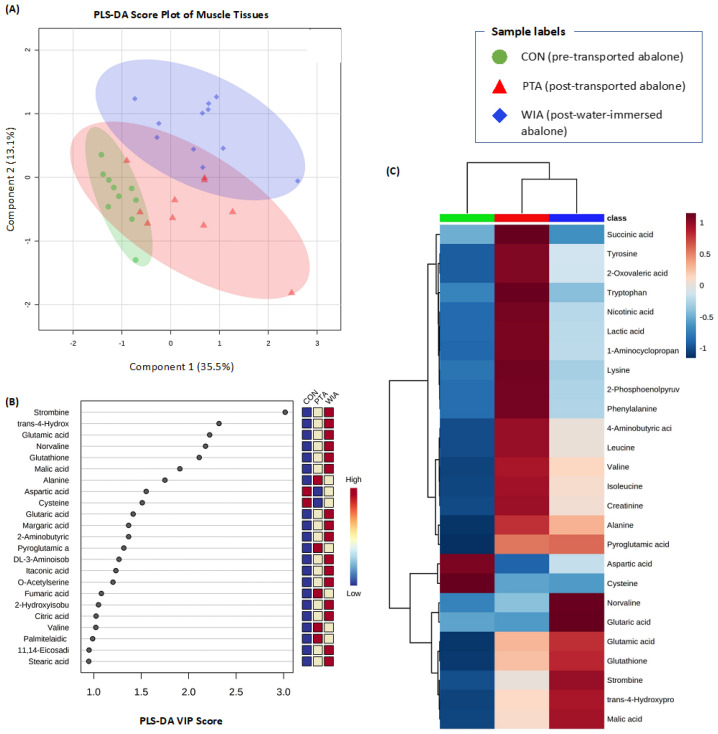
Effects of transport and re-immersion on the metabolite profiles of abalone muscle tissues. (**A**) PLS-DA score plots. (**B**) Relative abundance of metabolites with VIP scores greater than 1.0. (**C**) Heatmap of 26 metabolites that were different among treatments. The colour bars in the PLS-DA VIP score plot and heatmap show the relative abundance of metabolites (stronger blue: lower abundance; stronger red: higher abundance).

We identified 78 annotated metabolites in spectra of abalone muscle tissues. As with haemolymph, the majority of the metabolites in muscle samples were amino acids, fatty acids and organic compounds. PLS-DA score plots demonstrated a distinct distribution amongst treatments, especially between control abalone and post-water-immersed abalone (Figure 2A). There were some overlaps in PLS-DA score plots between post-transported abalone and the other two treatments (control and post-water-immersed abalone). The first two components made up 48.6% of the total variation. The cross validation of PLS-DA model via LOOCV of the first five components indicated a good prediction model with high scores of accuracy (0.87), R^2^ (0.96) and Q^2^ (0.81). There were 23 metabolites with VIP scores greater than 1, which were the most important classifiers (Figure 2B).

One-way ANOVA analysis revealed 26 metabolites that were different among the three treatments (*p* < 0.05). A heatmap of these metabolites clearly shows the differences in metabolite abundance for these treatments (Figure 2C). Compared to the control, post-transported abalone showed increased levels of organic compounds (e.g., succinic acid, lactic acid), amino acids (e.g., tyrosine, tryptophan, lysine) and other compounds (2-oxovaleric acid, pyroglutamic acid), but decreased levels of three amino acids (aspartic acid, cysteine, norvaline) and one organic compound (glutaric acid). Levels of norvaline and glutaric acid were not significantly different between control and post-transported treatments, but were found elevated in post-water-immersed abalone (Figure 2C). The metabolite profile of post-water-immersed abalone also showed high levels of many amino acids (e.g., norvaline, glutamic acid, glutathione), an amino acid derivative (pyroglutamic acid) and two organic compounds (glutaric acid, malic acid), but lower levels of aspartic acid and cystine compared to control abalone.

The pathway analysis of metabolite profiles of muscle tissues from pre-transport and post-transported abalone revealed 17 pathways with PIs greater than 0.01 in abalone muscle tissues affected by transport (Table 2). Among these, 13 pathways were identified as significantly different between the transport treatments (*p* < 0.05, number of hits ≥ 2). The remaining four pathways (*p* > 0.05 or number of hits < 2) were considered as only slightly affected by transport, including histidine metabolism, tryptophan metabolism, glycolysis/gluconeogenesis and fatty acid biosynthesis.

### 2.3. Biomarkers Associated with Transport Effects

Classical univariate receiver operating characteristic (ROC) curve analyses of post-transport treatment and control revealed 25 and 19 metabolites in haemolymph and muscle, respectively, that had area under ROC curve (AUC) values ≥ 0.9 (Table 3 and Table 4). Among these, there were 13 metabolites in haemolymph with AUC equal to 1, identifying them as putatively important haemolymph biomarkers associated with transport stress or recovery (Table 3). In muscle tissues, there were six biomarkers with AUC equal to 1 (Table 4). Among these, succinic acid was the only metabolite with AUC equal to 1 in both haemolymph and muscle tissues and showed increased levels in post-transported abalone compared to control animals.

## 3. Discussion

In this study, we report for the first time the use of a GC–MS-based metabolomics approach to assess the effects of live transport on the metabolism of abalone and the metabolic changes of these animals when re-immersed in water. Metabolite profiles of post-transported abalone compared to control (pre-transported animals) and post-water-immersed abalone revealed differences in the level of many metabolites in both haemolymph and muscle tissues. Most of these altered metabolites were energy production-related metabolites (e.g., amino acids and TCA intermediates), osmolytes (e.g., alanine) and anaerobic metabolism end-products (e.g., lactic acid, succinic acid).

The abalone that were transported in this study were starved for 4 days, which required animals to use energy reserves for basic metabolism and stress responses during this time. These responses were evident in the metabolite profiles of post-transported individuals which showed decreased levels of many amino acids in both haemolymph (aspartic acid, methionine, asparagine, leucine, phenylalanine, serine, 2-aminobutyric acid) and muscle (aspartic acid, cysteine, norvaline) samples. Amino acids are an important source of cellular energy metabolism in molluscs [31,32], and their decrease suggests that the animals had high energy demands. Decreases in amino acid concentrations have been demonstrated in metabolite profiles of molluscs in responses to different stressors, such as pathogens [23,33], hypoxia [34], heat and hypoxia [35], and harvesting and transporting [7]. In a recent study we also observed decreases of many amino acids in haemolymph of New Zealand green-lipped mussels (*Perna canaliculus*) after harvesting and air exposure [7]. Among these metabolites, aspartic acid decreased in both haemolymph and muscle tissues of post-transported abalone, as well as in the haemolymph of post-transported mussels [7]. The high demand for aspartic acid may reflect the important role of this metabolite for host metabolism during emersion stress. Indeed, aspartic acid is a constituent of most proteins and also plays an important role in the metabolism of nitrogen and neurotransmission [36]. Interestingly, there was a slight increase in aspartic acid levels in abalone that were placed back into water (re-immersed) (Figure 1 and Figure 2). This suggests that the level of aspartic acid could have the potential to be a biomarker for stress or health status of transported abalone (Table 1 and Table 2). In addition to aspartic acid, methionine and asparagine also increased modestly in re-immersed abalone, suggesting a partial recovery from transport stress.

An obvious challenge for abalone during transportation is the limited availability of oxygen when animals were emersed during transport, eliminating hydrostatic gill support and irrigation [12]. This is reflected by changes of many energy-related metabolites in post-transport abalone, including TCA cycle intermediates (2-oxoglutaric acid, succinic acid, malic acid, fumaric acid, isocitric acid, cis-aconitic acid) and anaerobic end-products (lactic acid, succinic acid, alanine). The TCA cycle is the key pathway to generate energy for all aerobic organisms. The disturbance or disruption of this pathway due to stress exposure or pathogen infections is often seen to result in the accumulation of metabolic intermediates in both vertebrates [37,38] and invertebrates [30,33,39], including abalone species [40]. In a comparable study, increases of many TCA cycle intermediates (succinic acid, malic acid and fumaric acid) were observed in the haemolymph of clams following 6 h of emersion [30]. Corresponding patterns of increases in citric, succinic, fumaric and malic acids were described in the haemolymph and hepatopancreas of cultured mussels after harvesting and storage in air for several hours [7]. In the present study, abalone were transported for 2 days without water, and we found an increase of 6 TCA cycle intermediates in haemolymph (2-oxoglutaric acid, succinic acid, fumaric acid, malic acid, isocitric acid, cis-aconitic acid) and 2 in muscle (succinic acid, malic acid). Unsurprisingly, pathway analyses also indicated that the TCA cycle was affected by transport stress (Table 1 and Table 2). The results are consistent with the disruption of aerobic metabolism via the TCA cycle due to the lack of oxygen availability when abalone were emersed for 2 days.

The disturbance of aerobic metabolism is further confirmed by the elevated levels of many anaerobic end-products in post-transported abalone, including lactic acid, succinic acid and alanine. Lactic acid is a well-known end product of anaerobic glycolysis [41,42]. Accumulation of lactic acid has been reported in different tissues of abalone [43,44,45,46] and other marine shellfish [7,30,47,48,49] under different anaerobic or stress conditions. Similar to lactic acid, accumulation of succinic acid and alanine as end-products of anaerobic metabolism has been demonstrated in many invertebrates [50,51,52]. Interestingly, the levels of lactic acid, succinic acid and alanine in both haemolymph and muscle of abalone decreased when animals were put back into the water. The biomarker analyses also revealed very high AUC values of lactic acid and succinic acid (Table 3 and Table 4), suggesting they could be reliable biomarkers for anaerobic metabolism and stress responses in abalone. Together, our findings suggest that the lack of oxygen during transportation disturbed energy metabolism and resulted in a shift from aerobic to anaerobic metabolism in abalone.

As soon as the animals were taken out of the water and placed in the box for transportation, they faced potential osmotic stress due to the absence of seawater and the potential for evaporative water loss. This is reflected in the elevated levels of many amino acids in metabolite profiles of haemolymph and muscle tissues from post-transported abalone compared to control animals (Figure 1 and Figure 2). Aquatic invertebrates, including molluscs, are able to use high concentrations of amino acids and derivatives of amino acids as organic osmolytes to balance internal and external osmotic gradients [53,54,55]. In the present study, the levels of amino acids were increased in post-transported abalone, but then decreased when the animals were put back into the water (Figure 1 and Figure 2). The pathway analysis identified many amino acid metabolism/synthesis processes affected by transport (Table 1 and Table 2). This may implicate the involvement of amino acids in osmoregulation of the host. Indeed, the haemolymph osmolality of many molluscan species was found to increase after aerial exposure [56,57,58]. Among these elevated amino acids, alanine is one of the most abundant osmolytes in molluscs and crustaceans [59]. Wiesenthal, Müller, Harder and Hildebrandt [60] found that alanine, together with proline and urea are the major organic osmolytes in the snail *Theodoxus fluviatilis* under hyperosmotic stress. These findings suggest that, alongside its potential role as an anaerobic end-product, alanine could be a biomarker for osmotic stress in molluscan species. In the present study, the biomarker analysis via AUC corroborates that alanine is a robust biomarker for transport stress in *H. iris* (Table 4).

The accumulation of amino acids in this study may also be the consequence of protein degradation due to oxidative stress and apoptosis. Oxidative stress due to the rapid increase of reactive oxygen species (ROS) in cells or tissues in response to stressors is known to cause oxidative damage to cells and tissues via degradation of DNA, proteins and lipids [61,62]. This process has been demonstrated in several recent studies of molluscs exposed to different stress conditions [28,30,33,39,63,64], including the rapid increase of ROS in clams under aerial exposure [30]. ROS is known to be mitigated via the glutathione pathway [28]. In addition to increases of metabolites in the glutathione metabolism pathway (cysteine, glutamic acid, glutathione, glycine, lysine, methionine, serine), fatty acids and amino acids were also accumulated in mussels after being harvested and stored in bags for many hours, presumably representing further damage caused by oxidative stress [7]. Similarly, we observed increases of many metabolites in the glutathione metabolism pathway in post-transported abalone, and the glutathione pathway itself was also identified as being activated by the transport stress. The oxidative stress caused by aerial exposure, in turn, could lead to cell apoptosis and irreversible tissue damage [65,66]. Increases in amino acids and fatty acids as a consequence of apoptosis-associated damage have been demonstrated in previous studies in molluscs [16].

One interesting observation in this study is the metabolite profile of reimmersed abalone, which showed reduced stress signatures compared to post-transport, emersed abalone. These include the decreases of TCA cycle intermediates and anaerobic end-products (lactic acid, succinic acid and alanine), and the increased level of some amino acids (Figure 1 and Figure 2). This indicates that the animals were showing significant signs of recovery from emersion stress after 2 days of reimmersion. However, there were still some differences between metabolite profiles of re-immersed abalone and the pre-transported controls, possibly due to the fact that the animals were not fed during the immersion period. A recent investigation on mussels showed some signatures of recovery in metabolite profiles after reimmersing in seawater [7]. These studies highlight the importance of immersion stages during live transportation of shellfish. Indeed, reimmersion remains an effective method for restoring metabolic perturbations and prolonging life for shellfish.

In conclusion, this study provides molecular-scale evidence of the nature of stress caused by commercial live transport. Live transport of abalone caused a disturbance in aerobic energy metabolism and evidence of osmotic stress, cell and tissue damage as a consequence of oxidative stress and apoptosis. These negative impacts related to transport stress were reduced when the animals were put back into water. This suggests that it is important to keep the animals in seawater for as long as possible before transport and placed back in water as soon as arrival at destination for recovery. Our findings provide important information that may guide industry decisions to update and refine the methods and systems for live transport of abalone and other shellfish.

## 4. Materials and Methods

### 4.1. Experimental Design

To test the effects of live transport on abalone, we collected 40 New Zealand black-footed abalone (*H. iris*) (440.4 ± 41.5 g; 131.3 ± 4.4 mm) from Ascotts, Chatham Islands, New Zealand (44°00′59.0″ S 176°23′11.7″ W) in March 2020. Ten of these animals were sampled in the field (‘control’ or pre-transported group). Shell length and live weight measurements were taken, followed by haemolymph extraction. A sterile syringe with a 25G needle was used to withdraw 0.5 mL haemolymph from the pedal sinus of each animal. The haemolymph sample was transferred into a cryovial and snap-frozen in liquid nitrogen. The animals were then dissected, and muscle tissue samples were collected into cryovials for freezing in liquid nitrogen. All samples were then transferred to dry ice for transport to the AUT laboratories, where they were stored at −80 °C prior to analysis.

The remaining 30 animals were transported from the field collection site in dry bins, covered with wet hessian to simulated live-harvest conditions. After the initial transport (1 h), the animals were placed in commercial holding tanks and held for two days, following normal commercial practice. The tanks received continuous flowing seawater at ambient sea temperature. After the two days, the animals were packed in 4 polystyrene boxes (10 animals per 50 L box; humid air ~6 °C) and airfreighted to Auckland. Upon arriving in the lab, abalone were kept in boxes for another day to stimulate a 48 h transport event. Then, 10 abalone from one box were sampled for metabolomics as described above. The remaining animals were placed into a seawater tank connected to a recirculating system for 2 days, mimicking a commercial resuscitation system at transport destination. Ten randomly selected animals from this batch were then sampled as described above. The time period from removal of abalone from the sea to placement in the live-holding tanks in Auckland was 6 days.

### 4.2. Metabolite Extractions and Derivatization

All chemicals used for metabolomics analysis were of analytical grade. Chloroform, sodium hydroxide, sodium bicarbonate, the amino acid mixture, and standard alkane mixture were obtained from Merck (Darmstadt, Germany). 2,3,3,3-d4-alanine, methyl chloroformate (MCF), pyridine and methanol were obtained from Sigma-Aldrich (St. Louis, MO, USA) and anhydrous sodium sulphate was sourced from BDH chemicals (Poole, UK).

Stored haemolymph samples were thawed on ice before placing 500 μL into a new vial with 20 μL of 10 mM d_4_ alanine, and re-stored at −80 °C. Then, samples were dried in a SpeedVac Concentrator (Savant SC250EXP) with a Savant RVT5105 refrigerated vapor trap (Thermo Scientific, Asheville, NC, USA) for 4 h (0 °C, vacuum ramp 3, 42 Torr/min). The muscle samples were freeze-dried overnight in a Christ Alpha 1–2 LD freeze dryer (Martin Christ Gefriertrocknungsanlagen GmbH, Osterode am Harz, Germany), and then ground into fine powder using a mortar and pestle. Approximately, 10 mg of muscle were weighed and transferred into 1.5 mL Eppendorf tubes. All samples (haemolymph and muscle) were extracted using the cold methanol–water solution, as previously described by Smart, Aggio, Van Houtte and Villas-Bôas [67] with modifications by Nguyen, Alfaro, Young, Ravi and Merien [39]. The extracted metabolites were dried in a Savant SC250EXP SpeedVac Concentrator as specified above, before derivatization using the methylchloroformate (MCF) method described by Smart, Aggio, Van Houtte and Villas-Bôas [67]. The derivatized samples were transferred to 2 mL amber GC–MS glass vials fitted with 150 μL inserts with bottom-springs (Sigma-Aldrich, St. Louis, MO, USA) for GC–MS analyses.

### 4.3. GC–MS Analysis and Quality Control

The metabolomics analyses were conducted using a gas chromatograph GC7890B coupled with a quadrupole mass spectrometer MSD5977A (Agilent Technologies, CA, USA), with a quadrupole mass selective detector (EI) operated at 70 eV. The system was equipped with a ZB-1701 GC capillary column (30 m × 250 μm internal diameter × 0.15 μm film thickness with a 5 m guard column) (Phenomenex, Torrance, CA, USA). The instrumental setup parameters for MCF derivatized samples were set according to Smart, Aggio, Van Houtte and Villas-Bôas [67]. The GC-oven temperature was initially held at 45 °C for 2 min, and then raised with a series of gradient increases including: increased at 9 °C/min to 180 °C, held for 5 min, increased at 40 °C/ min to 220 °C, held for 5 min; increased at 40 °C/min to 240 °C, held for 11.5 min; increased at 40 °C/min to 280 °C, held for a further 2 min. The interface temperature was set to 250 °C, the source was set at 230 °C and the quadrupole temperature was set at 150 °C. Samples (1 μL) were injected under pulsed splitless mode with the injector temperature set at 260 °C. Helium was used as the carrier gas and was held at a constant flow of 1 mL/min. The GC column was equilibrated for 6 min prior to each analysis. The mass spectrometer was operated in scan mode, starting after 6 min with a mass range 38–650 AMU at 1.47 scans/s and detection threshold of 100 ion counts.

Quality control (QC) samples were employed to ensure reproducibility of GC-MS measurements. The first QC samples were chloroform solvent and non-derivatized n-alkanes (C10-C40) that were performed to check the instrument. The alkane samples were also used to check Kovats retention index and create the calibration file. Secondly, four standard amino acid mixtures (20 µL, 20 mM) were similarly derivatized and measured following the protocol for samples. Thirdly, four blank samples and pooled samples containing 20 μL of 10 mM d_4_ alanine were extracted, derivatized and analyzed using the sample protocol as above. These QC samples were injected at the beginning and after every ten samples. Together, QC samples made up more than 30% of all injections.

### 4.4. Data Processing and Data Analysis

Raw spectra were processed using the Automated Mass Spectral Deconvolution and Identification System (AMDIS) which is a freeware extensively used in metabolomics (online software distributed by the National Institute of Standards and Technology, USA—http://www.amdis.net/, accessed on 14 April 2021). GC–MS data mining was carried out using the automated MassOmics R-based package [68]. Identification was performed using an in-house MS library with the minimum matching criterion of 70% based on both the MS spectrum of the metabolite and its respective retention time. Annotated metabolites were manually checked with ChemStation software (Agilent Technologies, Inc., Santa Clara, CA, USA) and AMDIS for the presence of contaminants. Repeats (based on ID number, match factor and retention time) and aberrant records were removed. Data were normalized to dried biomass and then to the internal standard (d_4_ alanine) to compensate for potential technical variations (e.g., variable metabolite recoveries) prior to data analyses.

All statistical analyses were performed using MetaboAnalyst 5.0-a web-based metabolomics data processing tool (https://www.metaboanalyst.ca, accessed on 14 April 2021). Data were first normalized via auto scaling (mean-centred and divided by the standard deviation of each variable) to make individual features more comparable. Chemometric analyses with supervised PLS-DA were used to identify natural groupings of all samples and assess the discrimination between abalone treatments. The PLS-DA model performance was validated using LOOCV, which was assessed via accuracy, R^2^ and Q^2^. The important classifiers were identified via the PLS-DA VIP scores. All metabolites with VIP score values greater than 1 were considered important for separation among treatments. Then, univariate data analysis with one-way analysis of variance (ANOVA) was applied to identify details of differences among sample groups. A heatmap of detected metabolites were also generated to visualize differences.

Pathway analysis was performed using MetaboAnalyst 5.0 as specified above. Quantitative enrichment analysis (QEA) using global test algorithm [69] and network topology analysis (NTA) using relative-betweenness centrality [70] were used to investigate functional relationships among the annotated metabolites for pathway analyses. The pathway library of *Drosophila melanogaster* (fruit fly) in the Kyoto Encyclopaedia of genes and genomes (KEGG) database [71] was used as the reference. Pathways involving one or more annotated metabolites that matched with the KEGG database with simultaneous QEA *p*-values < 0.05, false discovery rate (FDR) < 0.05, and NTA pathway impact (PI) scores > 0.0 were considered as potential primary pathways of interest.

The MetaboAnalyst 5.0 was used for biomarker analyses. Classical univariate receiver operating characteristic (ROC) analyses (using linear support vector machines) were performed to assess the specificity and sensitivity of this metabolite for biomarker models based on the area under the ROC curve (AUC). Metabolites with AUC values greater than 0.9 were considered as accurate biomarkers for stress associated with transport.

## Figures and Tables

**Table 1:** List of pathways in abalone haemolymph affected by transport treatment. Hits represent the total compounds of each pathway found in the samples.

Pathways	Total Compounds of the Pathway	Hits	*p*-Value	False Discovery Rate (FDR)	Pathway Impact Scores
Cysteine and methionine metabolism	32	6	0.00	0.00	0.50
Glycine, serine and threonine metabolism	30	6	0.00	0.01	0.61
Phenylalanine metabolism	7	2	0.00	0.01	0.38
Phenylalanine, tyrosine and tryptophan biosynthesis	4	2	0.00	0.01	1.00
Tyrosine metabolism	33	3	0.02	0.06	0.19
Glyoxylate and dicarboxylate metabolism	24	8	0.02	0.06	0.49
Citrate cycle (TCA cycle)	20	6	0.06	0.15	0.31
Alanine, aspartate and glutamate metabolism	23	8	0.09	0.18	0.68
beta-Alanine metabolism	14	3	0.09	0.18	0.28
Arginine and proline metabolism	31	4	0.10	0.18	0.36
Tryptophan metabolism	30	1	0.13	0.21	0.21
Glutathione metabolism	26	6	0.13	0.21	0.50
Arginine biosynthesis	12	6	0.13	0.21	0.23
D-Glutamine and D-glutamate metabolism	5	3	0.26	0.36	1.00
Fatty acid biosynthesis	43	2	0.29	0.36	0.02
Histidine metabolism	9	1	0.88	0.88	0.40

**Table 2:** List of pathways in abalone muscle that were affected by transport. Hits represent the total compounds of each pathway found in the samples.

Pathways	Total Compounds of the Pathway	Hits	*p*-Value	False Discovery Rate (FDR)	Pathway Impact Scores
Phenylalanine, tyrosine and tryptophan biosynthesis	4	2	0.002	0.004	1.000
D-Glutamine and D-glutamate metabolism	5	3	0.002	0.004	1.000
Alanine, aspartate and glutamate metabolism	23	8	0.000	0.000	0.682
Glycine, serine and threonine metabolism	30	5	0.002	0.004	0.608
Cysteine and methionine metabolism	32	6	0.010	0.014	0.502
Glutathione metabolism	26	6	0.000	0.001	0.496
Arginine and proline metabolism	31	5	0.004	0.006	0.430
Histidine metabolism	9	1	0.088	0.105	0.400
Phenylalanine metabolism	7	2	0.002	0.004	0.379
Glyoxylate and dicarboxylate metabolism	24	6	0.007	0.011	0.279
beta-Alanine metabolism	14	3	0.000	0.000	0.277
Citrate cycle (TCA cycle)	20	6	0.001	0.004	0.262
Arginine biosynthesis	12	6	0.000	0.001	0.229
Tryptophan metabolism	30	1	0.010	0.014	0.214
Tyrosine metabolism	33	3	0.011	0.015	0.187
Glycolysis/Gluconeogenesis	26	1	0.006	0.009	0.104
Fatty acid biosynthesis	43	2	0.186	0.197	0.017

**Table 3:** Haemolymph metabolites with AUC greater than 0.9, identifying them as putative biomarkers for transport stress. The decrease or increase shows the change of metabolites in post-transported abalone relative to the control animals.

Compounds	AUC	T-test *p*-Value	Log2 FC	Increase/Decrease
Asparagine	1.00	<0.01	− 3.03	↓
Methionine	1.00	<0.01	−2.63	↓
Aspartic acid	0.95	<0.01	−2.01	↓
Phenylalanine	0.99	<0.01	−1.24	↓
S-Adenosylmethionine	0.90	<0.01	−1.12	↓
Leucine	0.96	<0.01	−0.75	↓
4-Aminobutyric acid (GABA)	0.94	<0.01	−0.71	↓
Glyoxylic acid	0.95	<0.01	0.84	↑
Isocitric acid	0.95	<0.01	1.13	↑
Itaconic acid	1.00	0.01	1.17	↑
Malonic acid	0.95	<0.01	1.28	↑
Cysteine	0.96	<0.01	1.53	↑
Cystathionine	0.96	<0.01	1.69	↑
cis-Aconitic acid	1.00	<0.01	1.93	↑
DL-3-Aminoisobutyric acid	1.00	<0.01	2.02	↑
2-Oxoglutaric acid	1.00	<0.01	2.07	↑
beta-Alanine	0.97	<0.01	2.23	↑
Maleic acid	1.00	<0.01	2.30	↑
Fumaric acid	1.00	<0.01	2.61	↑
Glutaric acid	1.00	<0.01	2.78	↑
Malic acid	1.00	<0.01	3.42	↑
2-Hydroxybutyric acid	1.00	<0.01	3.48	↑
Tyrosine	0.98	<0.01	3.52	↑
Lactic acid	1.00	<0.01	3.84	↑
Succinic acid	1.00	<0.01	5.53	↑

**Table 4:** Muscle metabolites with AUC greater than 0.9, identifying them as putative biomarkers for transport stress. The decrease or increase shows the change of metabolites in post-transported abalone relative to the control animals.

Compounds	AUC	T-test *p*-Value	Log2 FC	Increase/Decrease
Aspartic acid	1.00	<0.01	−1.69	↓
Creatinine	1.00	<0.01	0.62	↑
Glutamic acid	1.00	<0.01	0.60	↑
Glutathione	1.00	<0.01	0.57	↑
Strombine	1.00	<0.01	2.59	↑
Succinic acid	1.00	<0.01	2.04	↑
2-Phosphoenolpyruvic acid	0.99	0.01	4.15	↑
Alanine	0.99	<0.01	0.98	↑
1-Aminocyclopropane-1-carboxylic acid	0.98	<0.01	0.96	↑
Citric acid	0.96	<0.01	0.44	↑
Nicotinic acid	0.94	<0.01	1.78	↑
Phenylalanine	0.93	<0.01	0.23	↑
Valine	0.93	<0.01	0.37	↑
Isoleucine	0.93	<0.01	0.33	↑
Leucine	0.93	<0.01	0.33	↑
Pyroglutamic acid	0.93	<0.01	0.47	↑
4-Aminobutyric acid (GABA)	0.92	<0.01	0.31	↑
Lactic acid	0.91	<0.01	2.59	↑
Tyrosine	0.91	<0.01	0.37	↑

## Data Availability

Raw data and output of the metabolite identification are available on reasonable request to the corresponding author, A.C.A. The data are not publicly available due to a requirement from the funding organization.
